# Editorial: Microbiotechnology Tools for Wastewater Cleanup and Organic Solids Reduction

**DOI:** 10.3389/fmicb.2021.631506

**Published:** 2021-02-18

**Authors:** Mayur B. Kurade, Mukesh Kumar Awasthi, Sanjay P. Govindwar, Byong-Hun Jeon, Dayanand Kalyani

**Affiliations:** ^1^Department of Earth Resources and Environmental Engineering, Hanyang University, Seoul, South Korea; ^2^College of Natural Resources and Environment, Northwest A&F University, Yangling, China; ^3^School of Industrial Biotechnology, Royal Institute of Technology, Stockholm, Sweden

**Keywords:** emerging contaminants (ECs), wastewater treatment, phytoremediation, phycoremedation, nutrient removal, biodegradation, bioremediation

Millions of people around the world lack access to safe water and suffer the consequences of unacceptable sanitary conditions due to contamination of water resources with various synthetic and geogenic compounds, which are being leached from agricultural, industrial, and domestic activities (Kurade et al., 2019; Xiong et al., 2021). The primary objective of a wastewater treatment plant is to reduce the concentrations of the pollutants to the level at which the discharge of the effluent will not adversely affect the environment or pose a health threat. Treatment of wastewater using bioremedial tools is an attractive approach because of their cost-effective and environmentally friendly properties. The modifications and improvements of existing biological treatment systems and/or addition of advanced technologies into wastewater treatment plants (WWTPs) is important to maximize the treatment efficiency. Along with the bacterial-mediated wastewater treatment, microalgae- and plant- based remediation is also of growing scientific interest (Rane et al., 2016; Huang et al., 2017; Vila-Costa et al., 2017). This Research Topic aims to gather the current advancements in microbial biotechnologies for wastewater treatment and biosolids reduction from urban and industrial wastewater. It includes eight original research articles that deal with related topics, ranging from novel methodology in quantifying the wastewater processes to the biologically facilitated removal and degradation of a diverse class of water pollutants.

The activated sludge process is a widely employed biological treatment of industrial and municipal wastewater. To achieve the highest treatment efficiency, it is important to monitor and maintain the optimum biomass concentration. Asgharnejad and Sarrafzadeh, proposed a cost-effective, high-throughput and errorless methodology of image processing and Red Green Blue (RGB) analysis for the quantification of activated sludge. Several essential parameters required for the deployment of such a method at large-scale industrial conditions are addressed.

The contamination of water resources with pharmaceutical contaminants has become a serious environmental problem in recent times. Xiong et al., utilized a green microalga, *Scenedesmus obliquus*, for the treatment of wastewater contaminated with doxylamine. Doxylamine exhibited negligible effect on *S. obliquus* and its biochemical characteristics, including pigments. As a result, *S. obliquus* exhibited the effective removal of doxylamine, chemical oxygen demand, and nutrients from the wastewater. Authors have shown the feasibility of using microalgae-based biotechnologies for wastewater treatment, but concluded that pilot-scale studies are needed for its implementation.

Swine wastewater has detrimental effects on the water quality and aquatic ecosystem due to the presence of high concentrations of organic compounds, nutrients, heavy metals, and antibiotics. Therefore, Dan et al., investigated an intermittent cycle extended aeration system (ICEAS) for the removal of nutrients from anaerobically digested swine wastewater. Authors observed that the performance of the ICEAS was superior to conventional sequencing batch reactor technology in terms of nutrient removal, and the effluent quality was within the discharge standard, suggesting that ICEAS is a promising technology for wastewater treatment.

Water pollution created by colored wastewater is a serious issue as it leads to ecological disturbances and reduces the aesthetic value of water resources. It is immensely important to eliminate the xenobiotic compounds responsible for coloration before they are released into the environment. In this view, two original research articles in this article collection emphasized the application of bacterial-mediated remediation of two different kinds of colored wastewaters. Amin et al., improved the decolorization of a model, textile azo dye, Direct Red 81, under microaerophilic conditions by optimizing the nutritional and environmental parameters through statistical models. They explored the metabolic pathway and the active involvement of laccase and azoreductase in dye degradation and confirmed that the retrieved products were non-toxic. The contribution of Abdulsalam et al., focused on the decolorization of palm oil mill effluent using bacterial treatment. The treatment of palm oil mill effluent has been a challenge due to its intense brownish color due to excessive concentrations of tannins, melanoidin, and lignin compounds. Authors optimized the influential parameters of decolorization, including inoculum size, initial color concentration, and treatment time, using Response Surface Methodology.

The contribution of Thongpitak et al. outlined the treatment of a herbicide- paraquat contaminated waters using biologically synthesized manganese oxide considering its widespread contamination throughout the globe in soil and water, including rivers and surface waters, and its further accumulation in the food chain. The manganese oxide synthesized from microalga, *Pediastrum duplex*, performed Fenton-like reactions to deliver 50% degradation of paraquat within three days. It indicates that biosynthesized manganese oxide is an environmentally friendly alternative catalyst that can be effectively applied in wastewater treatment systems.

Phenol is a ubiquitous pollutant that is frequently observed in wastewater. Biological treatment of phenol-containing wastewater is an effective approach, however the toxicity of phenol on microorganisms hinders the process efficiency. Wang et al. explored the phenol tolerance mechanism of *Candida tropicalis* through transcriptomic analysis and showed that *C. tropicalis* prevented cell damage through improvement of cell wall resistance, maintenance of intracellular protein homeostasis, high-fidelity DNA replication, and organelle integrity. The outcomes of this study would help in the genetic modification of yeast for improving their efficiency of phenol degradation.

The utilization of *Dendrobium* plants for the phytoremediation of wastewater are highlighted due to their cost-effectivity and ecological advantages. *Dendrobium nobile* is the only plant that can produce the natural bioactive compound dendrobine, which has potential medical significance. The article by Sarsaiya et al., determined the presence of an endophytic fungal strain in *D. nobile*, and evaluated the production of endophyte and dendrobine using the molecular method and analytical characterization, respectively. The potential dendrobine producer strain in *D. nobile* was identified to be *Trichoderma longibrachiatum*, which exhibited strong bactericidal activity against common pathogens, indicating its potential in microbiotechnology fields.

With this Research Topic, we presented some newly developed techniques to deal with pollutants in wastewaters with a consideration of a broad readership with interest in the bioremediation of wastewater. Although the provided technologies are very attractive in terms of their efficiency, further in-depth investigations are needed to implement these technologies at an engineered scale. The inclusion of the existing research gaps and recommendations for future research in this Research Topic will ensure a productive progression toward a bright future of bioremediation.

## Author Contributions

All the authors of this editorial article have made a significant, direct and intellectual contribution to the work, and approved it for publication.

## Conflict of Interest

The authors declare that the research was conducted in the absence of any commercial or financial relationships that could be construed as a potential conflict of interest.
